# Case report: life-threatening coronary artery spasm under transversus abdominis plane block in combination with general anesthesia

**DOI:** 10.1186/s12871-018-0616-3

**Published:** 2018-10-20

**Authors:** Wenping Peng, Shun Huang, Shuzhen Zhou, Ning Yang, Mingzhang Zuo

**Affiliations:** Department of Anesthesiology, Beijing Hospital, National Center of Gerontology, China, No.1 DaHua Road, DongDan, Beijing, 100730 China

**Keywords:** Transversus abdominis plane block, Coronary artery spasm, Perioperative period, Electrocardiogram

## Abstract

**Background:**

Many cases of coronary artery spasm (CAS) during general plus epidural anesthesia have been reported. But transversus abdominis plane(TAP) block in combination with general anesthesia has not been reported to be a cause of CAS, let alone a life-threatening CAS.

**Case presentation:**

In this case report, we present a case of a patient with CAS accompanied by ventricular fibrillation under general anesthesia with TAP block.

**Conclusion:**

Coronary artery spasm, even life-threatening CAS, may occur during TAP block in combination with general anesthesia.

## Background

Transversus abdominis plane (TAP) block is a regional technique for analgesia of the anterolateral abdominal wall [1].TAP block in combination with general anesthesia in abdominal surgeries is proved to be beneficial in articles [2–5]. So it is used in our hospital.

Coronary artery spasm (CAS) plays an important role in the pathogenesis of variant angina as well as ischemic heart disease [6]. In a patient with CAS, characteristic electrocardiogram (ECG) changes allow diagnosis of perioperative CAS [7]. Many cases of CAS during general plus epidural anesthesia have been reported [8–10]. However, a case of CAS under general anesthesia with TAP block is rare. Here we describe a life-threatening CAS in a patient that might have been induced by TAP block.

## Case presentation

**Fig. 1:**
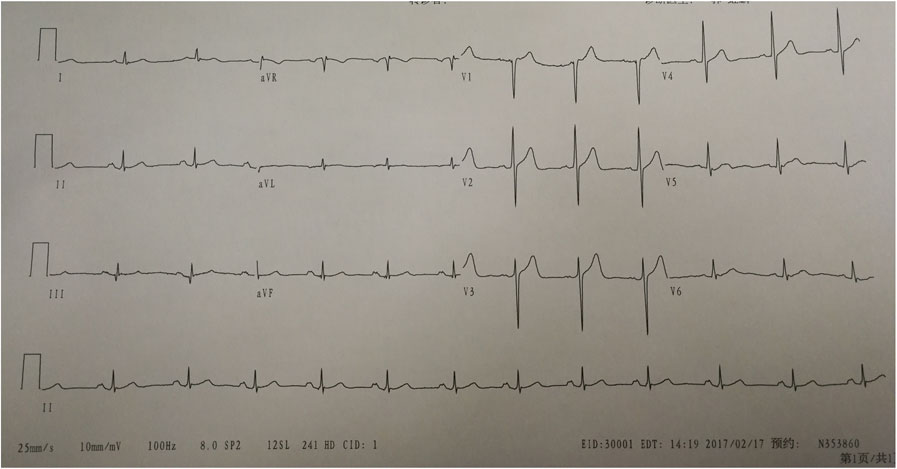
Preoperative ECG. Preoperative resting electrocardiogram(ECG) was within normal limits

A 43-yr-old, 66 kg, 175 cm man was to undergo gastrectomy for a tumor in the stomach. He had no history of hypertension, myocardial infarction,or angina pectoris,but with a smoking history(10 packs year).His physical examination was normal. Preoperative resting electrocardiogram (ECG) (Fig. 1)and echocardiogram(UCG) were within normal limits. Laboratory data revealed no abnormalities.

No premedication was given. After the patient^,^s arrival in the operating room, intravenous access was established. Lead II and V5 of the ECG were monitored. Blood pressure (BP) was 128/70 mmHg and heart rate(HR) 75 beats/min. An ultrasound-guided subcostal TAP block was performed bilaterally [1]. Each hemi abdomen was injected with 20 ml 0.3% ropivacaine to give a dual block from T_6_-T_9_. TAP block was uneventful without heart rate and blood pressure variations. After 30 min, general anesthesia was induced, then it was maintained with sevoflurane inhalation, target controlled infusion (TCI) remifentanil and given sufentanil and cisatracurium intermittently. The patient was mechanically ventilated with a tidal volume of 500 ml and respiratory rate of 10 breaths/min to maintain PetCO_2_ at 35–40 mmHg under end-tidal CO_2_ monitoring. Arterial BP was continuously monitored via a left radial artery catheter.

**Fig. 2:**
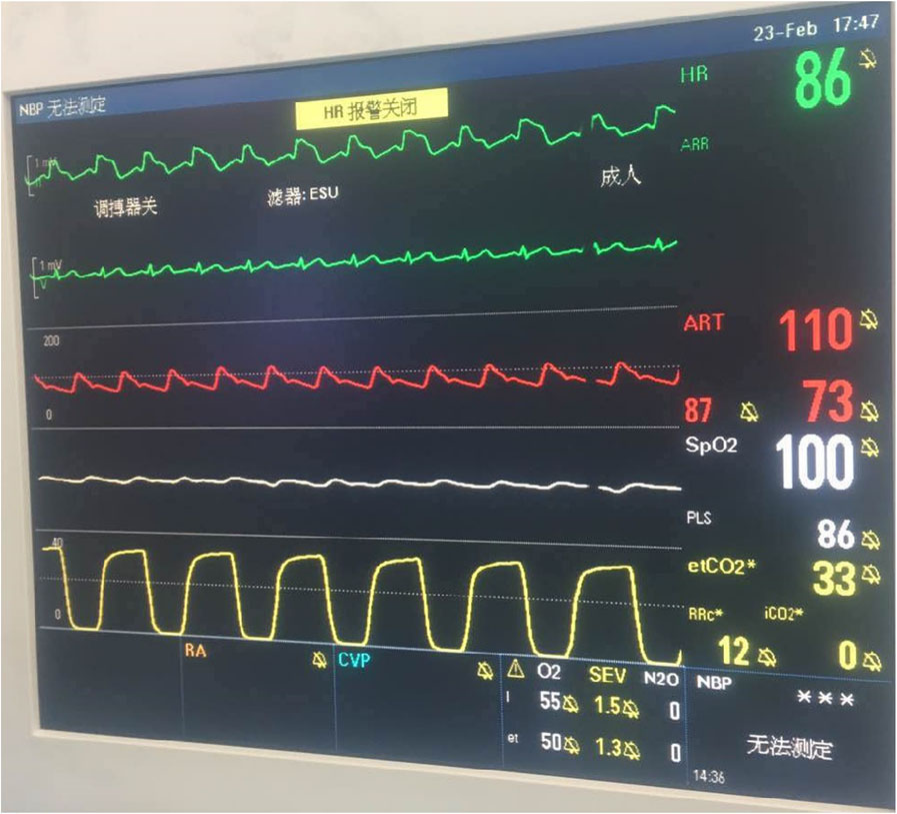
First ST segment elevation in lead II. ST segment elevation in lead II was firstly noted and lead V5 showed no abnormalities

**Fig. 3:**
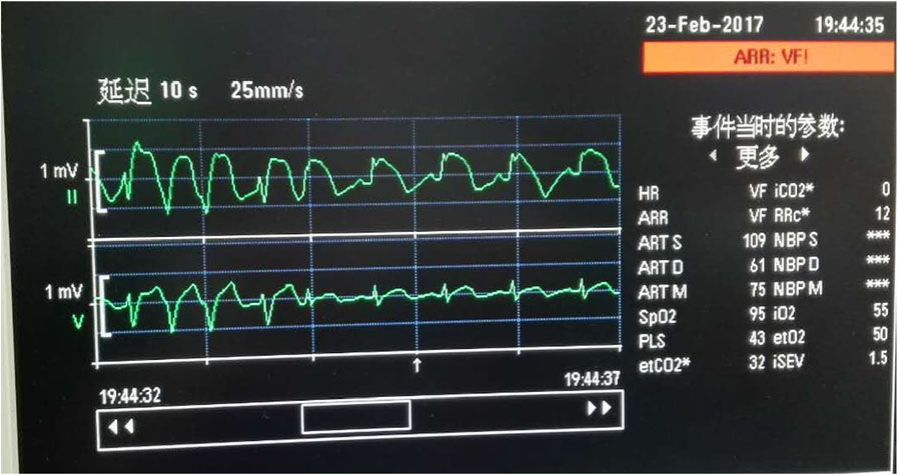
Second ST segment elevation in lead II. Second ST elevation in lead II and frequent premature ventricular contractions were recorded by the monitor

Two hours after start of the operation, when the surgeons were dissecting para gastric lymph node, ST segment elevation in lead II was noted (Fig. 2) and lead V5 showed no abnormalities. The change recovered abruptly without treatment 30 s later. When it happened, SpO_2_ was 100%, end-tidal sevoflurane concentration was 1.3% and no obvious hemorrhage. Except for this, the patient^,^s course during 4 h of operation was uneventful: BP was about 100/70 mmHg, HR about 70 beats/min, body temperature about 36.5 °C and estimated blood loss was less than 300 ml. Four hours after start of the operation, the arterial BP was 88/55 mmHg and aramine 0.4 mg was given intravenously. The BP increased to 110/65 mmHg without HR change. Approximately 5 min later, the ECG showed premature ventricular contractions and a marked ST segment elevation again (Fig. 3). Ventricular tachycardia and fibrillation were subsequently noticed with BP decreased to 32/14 mmHg, and electric defibrillation was initiated with repeated infusions of epinephrin. Within approximately 2 min, the ECG returned to sinus rhythm and BP gradually to normal. The patient remained hemodynamically stable for the remainder of the operation. Following the operation, he was transferred to the cardiac care unit (CCU) in our hospital. Serial ECGs and cardiac enzyme studies showed no abnormalities, thus ruling out myocardial infarction and CAS was diagnosed. The patient suffered no further cardiac attacks during his hospital stay. Consent was obtained from the patient to publish this case report.

## Discussion and conclusions

**Fig. 4:**
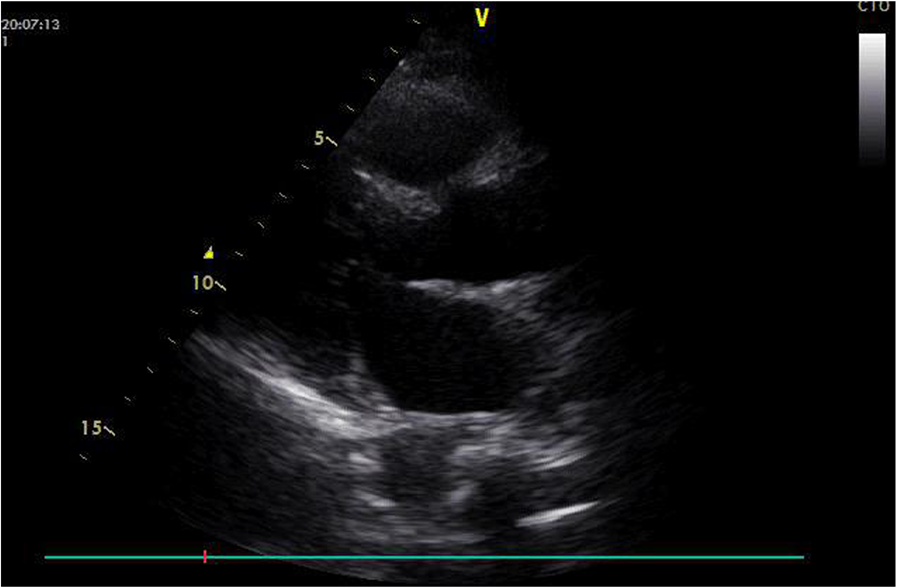
Trans thoracic echocardiography(TTE) just after ventricular fibrillation. TTE showed no air bubble in the heart

The episodes of ST segment elevation in the present case were most likely due to CAS, because the episodes were not preceded by increases in either HR or BP, and the postoperative ECGs and laboratory data were normal [7, 11]. Potential air emboli into the systemic circulation mimics CAS [10], but the trans thoracic echocardiography(TTE) just after ventricular fibrillation for the patient (Fig. 4) ruled out the possibility. The later sever CAS brought ventricular tachycardia and fibrillation.

The precise mechanism by which CAS occurs remains to be elucidated, but one of the important precipitating factors is the impairment of autonomic nervous system [12, 13]. Kenichiro Koshiba and Sumio Hoka noted that perioperative CAS occurred most frequently under inhalation general anesthesia combined with epidural anesthesia (46%) [10].Sympathetic excitation above the level of sympathetic blockade is thought to cause CAS associated with epidural anesthesia [14]. TAP block in combination with general anesthesia has not been reported to be a cause of CAS. The sympathetic nerves relevant to abdominal wall run into the transverse abdominis plane accompanying with thoracolumbar nerves [1, 15], the local anesthetic spread in this plane (TAP block) can block this part of sympathetic nerves. More importantly, local anaesthetic within that plane may spread posteriorly to the paravertebral space to block sympathetic nerves [16]. We performed TAP block bilaterally, so maybe sympathetic nerves in paravertebral space were blocked more widely. Reflex sympathetic activity involving the cardiac sympathetic nerves above the level of sympathetic blockade causes CAS, which is similar to the mechanism of epidural anesthesia causing CAS.

Vasopressors, such as dopamine, ephedrine, as well as other agents, were thought to have potentiated the occurrence of perioperative CAS [10]. However, the first episode of ST segment elevation in our patient was not associated with vasopressors. Maybe the aramine, α-stimulant, made the second episode of ST segment elevation worsen, but we did not believe this to be the direct cause. The management of CAS consists of administration of nitrates and calcium antagonists [17]. The first episode of ST segment elevation in our patient recovered abruptly without treatment, and we did not pay much attention to it to give nitrates or calcium antagonists. That may also result in the second episode of CAS worsen.

The definitive diagnosis of CAS can be made by angiographic demonstration of reversible coronary constriction [17]. But coronary angiography was not performed in our patient because we failed to obtain his consent and because he did not have risk factors such as hypertension, diabetes mellitus, or hyperglycemia [17].

In summary, we have described a rare case of CAS during TAP block in combination with general anesthesia in a patient under non-cardiac surgery. And the sever CAS resulted in life-threatening ventricular fibrillation.

## Abbreviations

BP: Blood pressure
CAS: Coronary artery spasm
CCU: Cardiac care unit
ECG: Electrocardiogram
HR: Heart rate
TAP: Transversus abdominis plane
TCI: Target controlled infusion
TTE: Trans thoracic echocardiography
UCG: Echocardiogram
